# A green approach of vat dyeing of cotton fabric with natural reducing agents

**DOI:** 10.1016/j.heliyon.2023.e19663

**Published:** 2023-09-01

**Authors:** Md Hossain Shahid Shrwardi, Shohag Chandra Das, Md Abdullah Al Mamun

**Affiliations:** a Department of Wet Processing Engineering, Bangladesh University Of Textiles, Bangladesh, Tejgaon, 1208, Dhaka; b Department of Dyes and Chemical Engineering, Bangladesh University of Textiles, Bangladesh, 1208, Tejgaon, Dhaka; c Department of Textile Engineering, Mawlana Bhashani Science and Technology University, Tangail, Tangail, 1902, Bangladesh

**Keywords:** Vat dyeing, Natural reducing agents, Ecofriendly, Green, Cotton dyeing

## Abstract

This study focuses on an eco-friendly approach to vat dyeing of cotton fabric using natural reducing agents and compare their performance with a conventional reducing agent. Natural reducing agents derived from pineapple barks, watermelon, and carambola extracts were investigated as alternatives. The reduction potential and FTIR of these natural extracted reducing agents were evaluated to assess their dyeing capability. Different colorimetric properties, color strength, colorfastness, bursting strength and pilling resistance of the dyed fabrics were analyzed. The environmental impact of the dyeing process was assessed by measuring parameters such as BOD, COD, TDS, DO and pH values. The results indicated that the natural reducing agents exhibited comparable reduction potential to sodium dithionite. Colorimetric analysis revealed that the optimal concentrations of the reducing agents for dyeing were 500–600 ml/l. The carambola extract showed the best colorimetric properties among the three reducing agents tested. The study demonstrates the potential of natural reducing agents as eco-friendly alternatives for vat dyeing of cotton fabric, contributing to sustainable and green textile dyeing processes.

## Graphical abstract

**Figure:**
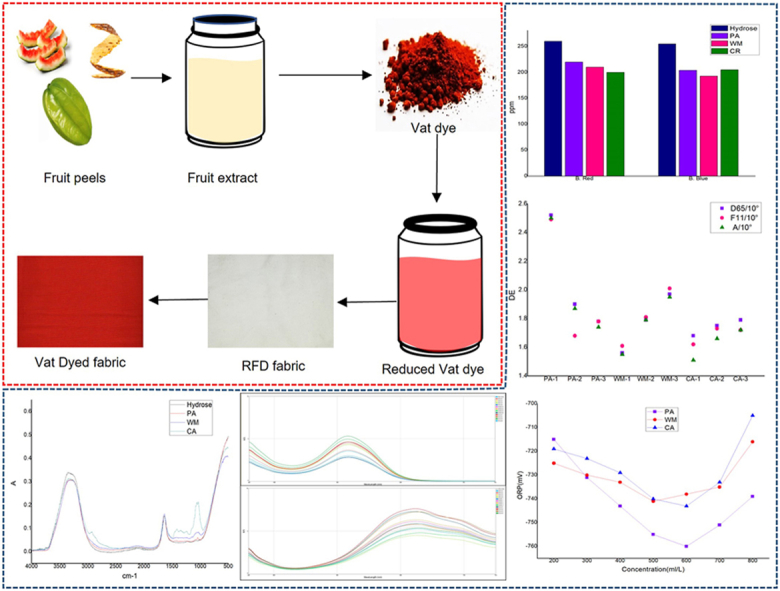

## 1 Introduction

Vat dyeing is one of the most popular methods of textile dyeing, especially for cotton fabrics, due to its ability to produce vibrant color and excellent colorfastness. Vat dyes are divided into two types: indigoid vat and anthraquinonoid vat [1,2]. The process contains the transformation of a water-insoluble dye to its water-soluble form by reduction, allowing it to penetrate and color the fibers, then dyeing and finally oxidation to make color water insoluble again [3]. Here, the reducing agent plays a crucial role in the reduction of vat dyes and making it available for the dyeing process [4]. It, also called vatting, makes the dyes to be capable of forming a chemical bond with the textile fibers, thereby imparting color [5].

Sodium dithionite (Hydrose) is a popular reducing agent for vat dyeing which is not eco-friendly and sustainable to nature because of its production of sulfite, sulphate, thiosulphate,and toxic sulfur in residue [6,7]. For this purpose, excessive amounts of hydrogen peroxide and alkali are needed for the treatment of Vat dyed effluents, which is not environmental friendly and mostly costly [8,9]. However, Natural reducing agents are often preferred, typically derived from plant-based materials, for their reducing properties as well as being biodegradable, renewable, and no for harmful by-products [10]. Although, some drawbacks hold them from extensive industrial applications such as Lower Reducing Power, Sensitivity to Environmental Factors, cost and Economics, availability, Color Variation etc(Shin, Choi, and Yoo 2014).

The pineapple (Ananas comosus) is a fruit of the Bromeliaceae family, Carambola (Averrhoa Carambola), is a species of Oxalidaceae family and Watermelon is a fruit of the cucurbitaceous family. The extracts of all of these three extracts have reducing capacity as the reduction potential should be near about–715 mV to −760 mV for a proper reduction of vat dyes [11,12].

In order to mitigate the environmental impact of hazardous reducing agents, several studies were conducted with burnt Ajveh [13], alkaline iron (II) salt (FeSO4) [14], Glucose [7]. monosaccharides (d-glucose, d-fructose, and d-galactose) and reducing disaccharides (lactose and maltose) [15], banana peel extract [16], Mao berry extract(Saikhao et al., 2021), Date palm, Banana and Apple extract [17]. Some metals were also used as reducing agents such as: zinc metal powder (2ownwm) and a combination of zinc metal powder (1.5% owf) and hydro-sulphite (0.5% owf) [18]. (zinc 2.0% owf

ferrous sulphate 2.0% owf, zinc + hydrosulphite 1.0% + 1.0% owf, and ferrous sulphate + hydrosulphite 1.0% + 1.0% owf) [19].

However, as per author knowledge, the reduction potential as well as economic factors have always been a matter of concerns for most of the previous studies as it affects greatly on the dyeing capability of vat dyes [20]. In the thinking of the solution of these problems, this study was conducted on the development of green and ecofriendly method for vat dyeing with three natural extracts as reducing agents.

For this purpose, Pineapple barks, Water melon and Carambola extracts were used to dye cotton knit fabric with two different types of vat dyes. The colorimetric properties, color strength (k/s), colorfastness, bursting strength and pilling resistance of the dyed fabrics was conducted and compared to hydrose. BOD, COD, TDS, pH values will also be measured to indicate the environmental impact at the end process. Oxidation Reduction Potential measurement& FTIR analysis will be used to examine functional groups of natural reducing agents in this eco-friendly vat dyeing process.

## 2 Materials and methods

### 2.1 Materials

Ripe Pineapple barks (PH-3.26), Water melon (PH-3.88) and Carambola (PH-2.06)were collected from the local markets Dhaka, Bangladesh. 100% cotton knit single jersey bleached fabric of 150 GSM has been used for vat dyeing. Bezathren Red FBB EPS and Bezathren Blue RS EPS (CHT- Switzerland) have been used in powder form as vat dyes. All the auxiliaries have been collected from WPE Laboratory, Bangladesh University of Textiles. The whiteness index of this fabric was 67.79 and tint −1.26,YI 6.52 before dyeing. its Yarn Count-28Ne (s/1) Stitch length −2.70 mm Dia-26″, inch Gauge −24” inch Finishing dia −26.5 inch.

### 2.2 Preparation of natural reducing agents

The raw materials were cleaned and cut into small pieces. Then blended with blender and squeezed by mesh/nylon fabric. Finally, the extracts were filtered by filtration paper and stored at bottle in a refrigerator. The stock solution was made by 60% raw extract and 40% water for the next processes.

### 2.3 Characterization of reducing extracts

The extracted reducing agents were confirmed by comparing with existing reducing agent, Hydrose-(Na_2_S_2_O_4_). FTIR spectra analysis for functional group and ORP measurement for reduction potential analysis.

#### 2.3.1 FTIR analysis

Fourier transform infrared (FTIR) spectra of the solid raw materials and liquid extracts were conducted with FTIR spectrometer, PerkinElmer Inc. USA, UATR Two spectrum, UK using LiTaO_3_ detector with resolution of 1 cm -^1^, with a range of 4000- 450 cm -^1^ at DCE Lab, BUTEX and compared with standard spectra of hydrose.

#### 2.3.2 Oxidation Reduction Potential (ORP) measurement

ORP of all the extracts were measured by both HI 98201 ORP meter, Hanna and AD14 ORP/PH meter, Adwa. At first, Bezathren Red FBB EPS was used for each natural reducing agents with seven different concentrations (200 ml/l,300 ml/l, 400 ml/l, 500 ml/l, 600 ml/l, 700 ml/l, 800 ml/l). the highest ORP for each agent was determined and the corresponding concentrations were used for the rest two dyes, Bezathren Blue RS EPS and Bezathren Yellow FBB EPS. Finally, the results were compared with hydrose at10 g/l.

### 2.4 Dyeing of cotton fabric

The dyebaths for vatting were prepared with required vat dyes (0.5%, 1%and 2% owf) with wetting agent (1 g/l), sequestering agent (2 g/l), and levelling agent(2 g/l) and mixed in hot water (50 °C). Natural reducing agents with four different concentrations (400 ml/l, 500 ml/l, 600 ml/l, 700 ml/l) for reduction and sodium hydroxide (38° Be-70 ml/l) for the solubilization were added and ran the dye bath until the vatting of the dyes took place over a period of about 45min at 55 °C. After that, 10 g/l Glauber salt was added to the reduced solution, followed by pretreated cotton fabric and dyed for 60 min at 65 °C at 1: 20 liquor ratio. Then the bath was then dropped, oxidized with hydrogen peroxide (8 g/l) at 60 °C for 20min and air oxidation 10 min, followed by reduction clearing with natural reducing agent (50 ml/l) and Caustic Soda (2 g/l). Finally, hot rinse, washing, soaping at 98^0^c temperature and cold wash were done. The same procedure was followed for hydrose (5 g/l, 7.5 g/l, 10 g/l).

### 2.5 Colorimetric properties analysis

The CIELab, CIELch values and the K/S values of 2% vat dyed samples were measured with spectrophotometer (Data color 650) with three different light sources (D65-10, TPL4 and A-10) illumination and 10° observer angle. 4 folds were used and an average of two readings of each sample was taken. Kubelka–Munk formula was used to determine color strength values from the reflectance.

### 2.6 Dyeing evenness check

Dyeing evenness of all 2% red and blue dyed samples were analyzed by data color machine at Orient chem tex. Ltd, Dhaka.

### 2.7 Colorfastness test

Colorfastness of the dyed samples with all the reducing agents were measured in different laboratories in Bangladesh University of Textiles. The wash and rubbing fastness were conducted with the standard method ISO 105-C03 and ISO 105-X12:2001(E)with 670 HD crock master (James Heal) at Orient chemical Tex. Ltd, Dhaka. The perspiration fastness and light fastness were conducted with the standard method ISO 105-E04:2013 and ISO 105 B02:2013on Q-SUN (model Xe −2) Xenon Arc Light fastness machine for 72 h at WPE Lab, BUTEX. Finally, the evaluation had been done with gray scale (1–5) and blue scale (1–8) both for fading and staining.

### 2.8 Bursting strength test

The bursting strength of the fabrics were conducted at TTQC laboratory, Bangladesh University of Textiles with bursting strength tester, P-10000m229B, SDL Atlas, USA.

### 2.9 Pilling resistance test

The pilling test has been conducted with Martindale Pilling Tester, model: 1605 MIDI-MARTINDALE. The best samples among four of each reducing agents were used depending on ORP and K/S values.

### 2.10 Ecological performance measurement

Various ecological performance of effluent produced from natural reducing agents and hydrose were tested at the Hohenstein Laboratories Bangladesh limited, Dhaka using reference methods of the American Public Health Association (APHA) [21]. These are BOD (APHA 5210B), COD (APHA 5220B), TDS (APHA 2540C), D.O. (APHA 4500OG).

## 3 Result and discussion

### 3.1 FTIR analysis

**Fig. 1:**
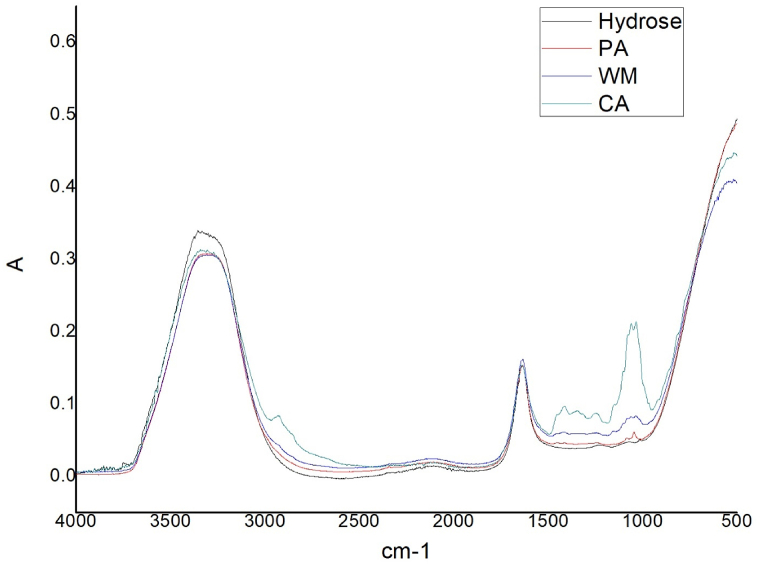
(a) FTIR spectra of the fruit extracts and.

The infrared spectra Fig. 1clearly shows that the peaks of hydrose and all other natural reducing agent are very identical which indicates the similar chemical group in all the natural reducing agents to hydrose. Here, all natural reducing agents have peaks at near 3355 -3500 cm^−1^ which indicates –OH (hydroxyl group), the peak found at 1635 cm^−1^ indicates C=O (Carbonyl group) and 1000-1200 cm^−1^ indicates C–OH stretch. These similarities strongly suggest that all those natural products are potential reducing agents [17]. had also explained the fact.

### 3.2 Oxidation Reduction Potential (ORP)

**Fig. 2:**
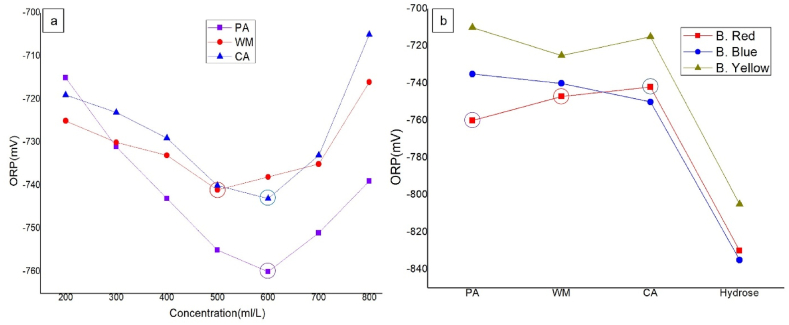
Oxidation Reduction Potentials (ORP) of the extracts for bringing suitable for reducing agent.

Fig. 2a shows that the ORP values for all natural reducing agents were in a good range (−705 mV–760 mV) for Bezathren Red FBB EPS dye at different concentrations. Among all the values, the highest ORP for each reducing agents were taken (−760 mV for PA at 600 ml/L, −741 mV for WM at 500ml/and −743mV for CA at 600ml/) and corresponding concentrations were applied for other two dyes. In Fig. 2b, the values for hydrose were highest in the case of all dyes but still all the natural reducing agents showed a good value. The values indicated the enough ORP of natural reducing agents to be used as reducing agent(Saikhao et al., 2022). also fund their the ORP value for Mao berry near this range which was comparable to existing reducing agents and most of the time the value ranges between −600 and −700mV [15].

### 3.3 Colorimetric properties analysis

**Fig. 3:**
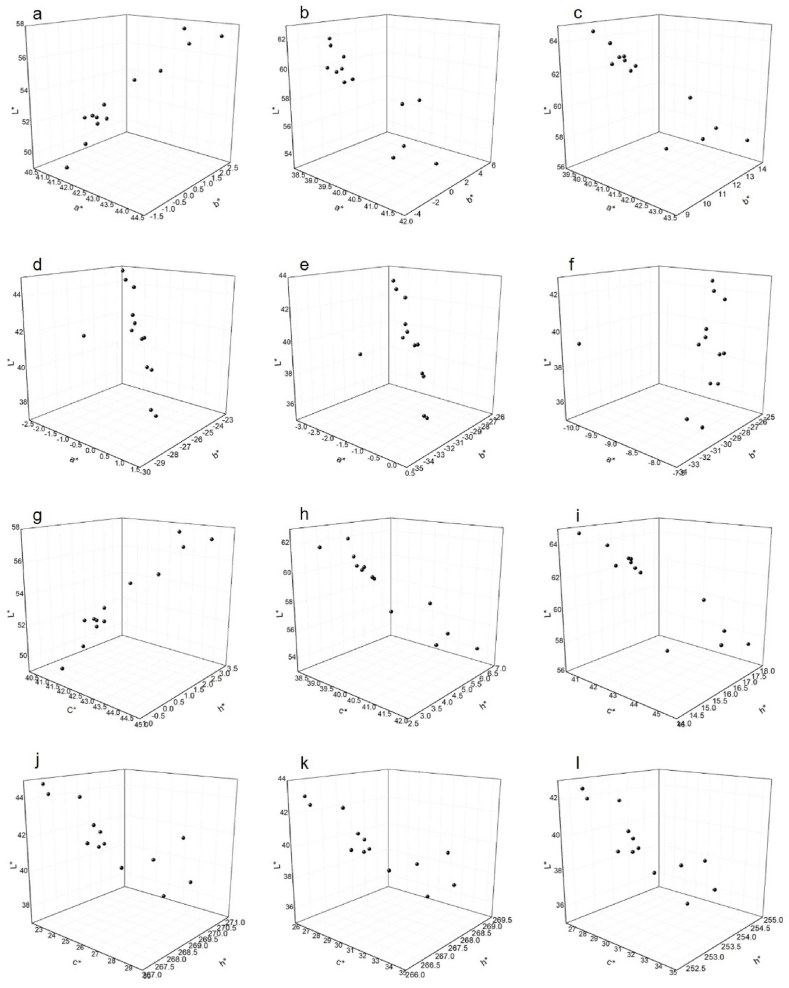
The colorimetric properties (L*a*b* and L*c*h*) of 2% vat dyed fabric at different.

Fig. 3 shows the effect of different reducing agents and their application amount in vatting which effects on final dyeing. The graphs summarize that the highest color depth for red dye at 2% was carambola at 600 g/l the value changes a bit for different light sources (Fig. 3-a, b, c, g, h, i) but the serials are maintained. For blue dyes at 2%, the values of a and b became negative, because of the color of the dye (Fig. 3- d, e, f, j, k, l). Here the lower a*- b* values indicate better dyeing. The L*c*h* values also indicate the similar idea.

**Fig. 4:**
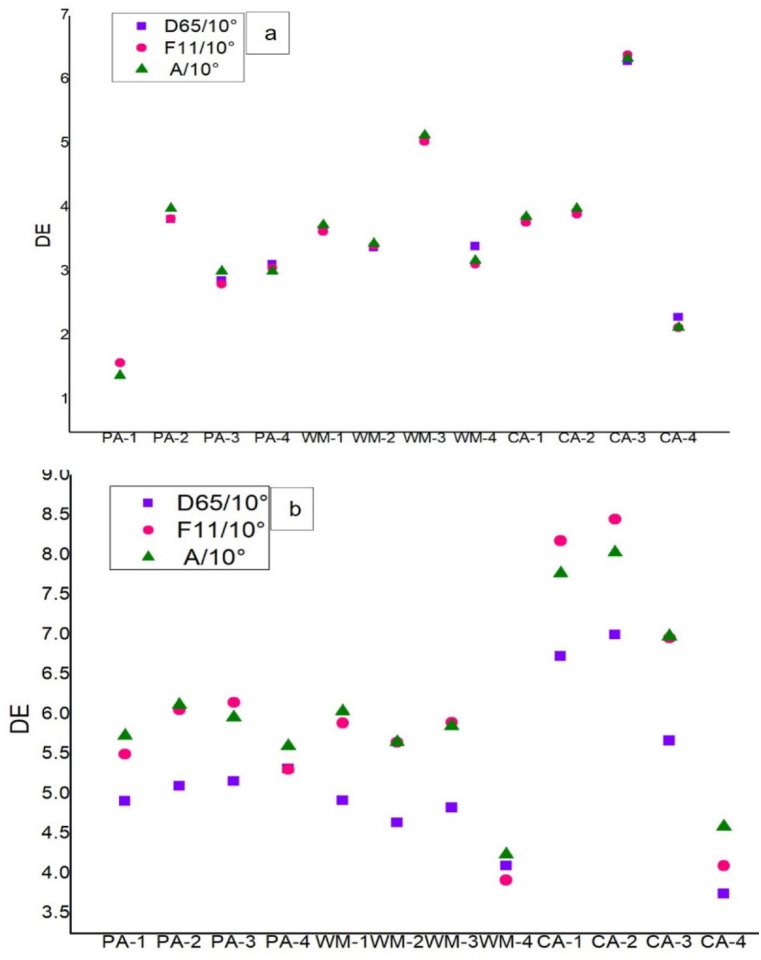
Color difference of the dyed fabrics with hydrose in different light source; a, - B. Red and b, - B. Blue for 2% dye shade.

The general outcome shows that 500 and 600 ml/l of the reducing agents work best for dyeing where 400 and 700 ml/l work comparatively lower (Fig. 4-a, b). The fact can be explained by the reason that the reducing agent works best up-to a specific concentration. Before and after of that specific concentration, the reduction capacity changes. Although the highest reducing power was for pineapple, but carambola showed better colorimetric properties. It can be explained by the fact that dyeing depends on many other factors [22]. So the effect of the amount of reducing agents indicate that the reducing agents not only impact on the dye ionization but also the number of leuco molecules in the dye bath which actually effects on the final dyeing (Saikhao et al., 2022).

light sources (a, d, g, j- D65/10, b, e, h, k-F11/10 and c, f, I, l- A/10) and two different dye class (B.Red-a, b, c, g, h, i and B. Blue-d, e, f, j, k, l)

The color difference in Fig. 4 supports the facts of dyeing performance of the fabric compared to conventional hydrose. Here the combined values of L*a*b* show the color difference values.

### 3.4 Dyeing evenness check

**Fig. 5:**
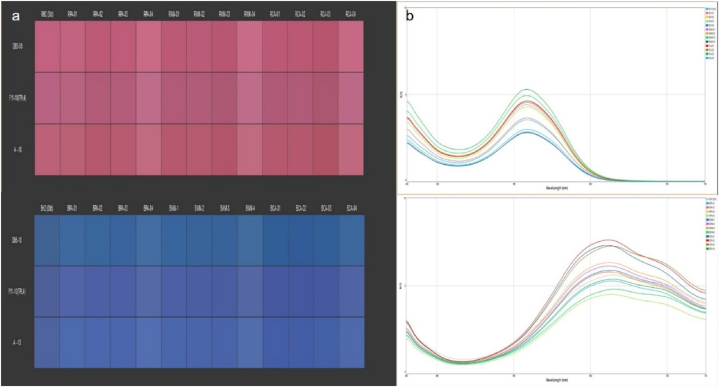
(a) Scanning images of dyed fabric to check the color depth with different reducing agents at different concentrations and (b) Color strength(K/S) of the dyed fabrics with different reducing agents at different concentrations for all 2%.

The scanning images shown in Fig. 5a, the color depth of the dyed fabrics. Here the highest depth for red dye was carambola at 600 ml/L, then watermelon at 600 ml/L, and again carambola at 500 ml/L as the third depth. The highest position for pineapple was 6th at 600 ml/L. The significant factor is the position of hydrose dyed fabric which is 11th among the 13 samples.

In the case of blue dyes, the highest depth was for carambola at 600 ml/L, then carambola at 700 ml/L, and carambola at 500 ml/L. The highest depth for pineapple was 4th position at 600 ml/L and 5th for watermelon at 600 ml/L. Here, similarly, the position for hydrose was 9th among 13 samples. All the findings also align with the color strength(K/S) values of the samples in.

Fig. 5b the highest color strength values were 5.3 and 7.7 for B red and B Blue respectively. The range of K/S values were compatible with previous findings to make sure for better dyeing [17].The visuals show that the dyed fabrics have good color property and suitable for being applied as potential reducing agents.

### 3.5 Colorfastness

Table 1 shows that the vat-dyed fabrics with different natural reducing agents have comparable wash fastness to hydrose. All the samples showed excellent value (5) for fading and most of the samples showed outstanding (5) fastness to wash and a few showed excellent (4/5). There is no change in fastness due to the change of shade % and hydrose shows excellent values for all concentrations and shade%.

**Table 1:** color fastness to wash.

	Bezathren Red FBB EPS								Bezathren Blue FBB EPS						
		Fading	Staining						Fading	Staining					
	Shade%		Acetate	Cotton	Nylon	Polyester	Acrylic	Wool		Acetate	Cotton	Nylon	Polyester	Acrylic	Wool
**HD**	**2%**	5	5	5	5	5	5	5	5	5	5	5	5	5	5
**PA**		5	5	4/5	5	5	5	5	5	5	4/5	5	5	5	5
**WM**		5	5	5	5	5	5	5	5	5	5	5	5	5	5
**CA**		5	5	4	5	5	5	5	5	5	4	5	5	5	5
**HD**	**1%**	5	5	5	5	5	5	5	5	5	5	5	5	5	5
**PA**		5	5	4/5	5	5	5	5	5	5	5	5	5	5	5
**WM**		5	5	5	5	5	5	5	5	5	5	4/5	5	5	5
**CA**		5	5	4	5	5	5	5	5	5	5	4	5	5	5
**HD**	**0.5%**	5	5	5	5	5	5	5	5	5	5	5	5	5	5
**PA**		5	5	4/5	5	5	5	5	5	5	5	5	5	5	5
**WM**		5	5	5	5	5	5	5	5	5	5	4/5	5	5	5
**CA**		5	5	4	5	5	5	5	5	5	5	4	5	5	5

The perspiration fastness in Table 2 of natural reducing agent dyed fabric also showed comparable values with hydrose similar as wash fastness, all the fading values showed excellent ratings (5) and most of the excellent values for each shade % indicated the suitability of the extracts as reducing agent.

**Table 2:** Color fastness to perspiration.

		Bezathren Red FBB EPS								Bezathren Blue FBB EPS						
			Fading	Staining						Fading	Staining					
		Shade%		Acetate	Cotton	Nylon	Polyester	Acrylic	Wool		Acetate	Cotton	Nylon	Polyester	Acrylic	Wool
**HD**	**Alkaline**	**2%**	5	5	5	5	5	5	5	5	5	5	5	5	5	5
	**Acidic**		5	5	5	5	5	5	5	5	5	5	5	5	5	5
**PA**	**Alkaline**		5	5	4/5	5	5	5	5	5	5	5	5	5	5	5
	**Acidic**		5	5	5	5	5	5	5	5	5	5	5	5	5	5
**WM**	**Alkaline**		5	5	4/5	5	5	5	5	5	5	5	5	5	5	5
	**Acidic**		5	5	5	5	5	5	5	5	5	5	5	5	5	5
**CA**	**Alkaline**		5	5	4	5	5	5	5	5	5	5	5	5	5	5
	**Acidic**		5	5	5	5	5	5	5	5	5	5	5	5	5	5
**HD**	**Alkaline**	**1%**	5	5	5	5	5	5	5	5	5	5	5	5	5	5
	**Acidic**		5	5	5	5	5	5	5	5	5	5	5	5	5	5
**PA**	**Alkaline**		5	5	5	5	5	5	5	5	5	5	5	5	5	5
	**Acidic**		5	5	5	5	5	5	5	5	5	5	5	5	5	5
**WM**	**Alkaline**		5	5	4/5	5	5	5	5	5	5	5	5	5	5	5
	**Acidic**		5	5	4/5	5	5	5	5	5	5	5	5	5	5	5
**CA**	**Alkaline**		5	5	5	5	5	5	5	5	5	5	5	5	5	5
	**Acidic**		5	5	5	5	5	5	5	5	5	5	5	5	5	5
**HD**	**Alkaline**	**0.5%**	5	5	5	5	5	5	5	5	5	5	5	5	5	5
	**Acidic**		5	5	5	5	5	5	5	5	5	5	5	5	5	5
**PA**	**Alkaline**		5	5	4/5	5	5	5	5	5	5	5	5	5	5	5
	**Acidic**		5	5	5	5	5	5	5	5	5	5	5	5	5	5
**WM**	**Alkaline**		5	5	4/5	5	5	5	5	5	5	4/5	5	5	5	5
	**Acidic**		5	5	5	5	5	5	5	5	5	5	5	5	5	5
**CA**	**Alkaline**		5	5	4	5	5	5	5	5	5	5	4	5	5	5
	**Acidic**		5	5	5	5	5	5	5	5	5	5	5	5	5	5

The rubbing fastness values in Table 3 were comparatively lower than the other two and the wet rubbing was less than dry rubbing due to higher frictional coefficient between fabric and crocking cloth. Most of the light fastness values were ranging from very good (6) to excellent (7) without the hydrose which is always excellent(7).Al the fastness values indicates the suitability of these natural reducing agents for vat dyeing.

**Table 3:** color fastness to rubbing and light.

		Rubbing Fastness				Light Fastness					
		Bezathren Red FBB EPS		Bezathren Blue FBB EPS		Bezathren Red FBB EPS			Bezathren Blue FBB EPS		
	Shade%	Dry	Wet	Dry	Wet						
**HD**	**2%**	5	4/5	5	4/5	7			7		
**PA**		5	4/5	5	4/5	6	6/7	6/7	6		6/7
**WM**		4/5	4/5	5	4	6	6	6/7	6	7	6/7
**CA**		5	4	4/5	4	6	6	6/7	6	7	6/7
**HD**	**1%**	5	4/5	5	4	7			7		
**PA**		5	4/5	4/5	4	6/7	6	6/7	6/7	6	6/7
**WM**		4/5	4	4/5	4	6	6	6/7	6	6	6/7
**CA**		5	4/5	4/5	4/5	6	6/7	6	6	6/7	6/7
**HD**	**0.5%**	5	5	5	4/5	7			6		
**PA**		5	4/5	5	4/5	6	6/7	6/7	6	6	6/7
**WM**		5	4/5	5	4/5	6	6/7	6/7	6	6	6/7
**CA**		4/5	4	5	4	6	6/7	6/7	6	6	6/7

### 3.6 Bursting strength test

**Fig. 6:**
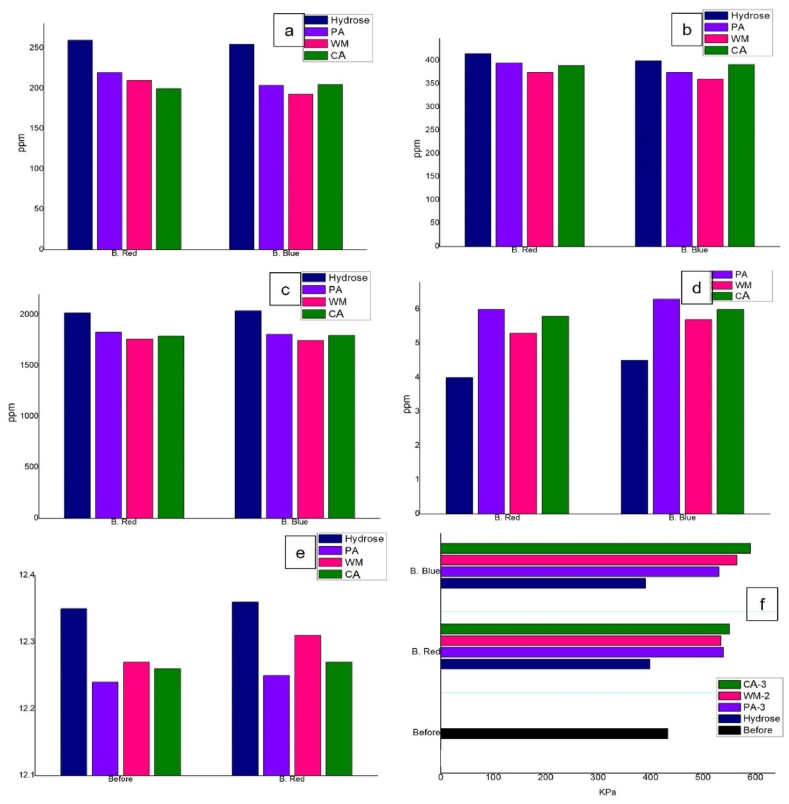
Ecological performance of the effluent produced from dyed fabric with different reducing agents (a) BOD, (b) COD, (c)TDS, (d)DO, (e) pH and f Bursting strength of the dyed fabrics with different reducing agents.

The change in bursting strength of the dyed fabrics with natural reducing agents was noticeable, (Fig. 6f). The values had been increased after dyeing in the case of both dyes where hydrose showed a reduction. The before strength was 433 KPa which was reduced to 398 and 390 for B. red and B. blue respectively. On the other hand, carambola showed the highest increment and watermelon as well as pineapple on the 2nd highest position corresponding for both dyes. The result indicates the increment in elastomeric properties of the dyed fabric due to natural reducing agents where the application of hydrose should be discouraged [23]. Moreover, the application of sodium hydroxide during the vatting process may be worked as a mercerizing agent which increased the bursting strength of the fabric.

### 3.7 Pilling resistance test

**Fig. 7:**
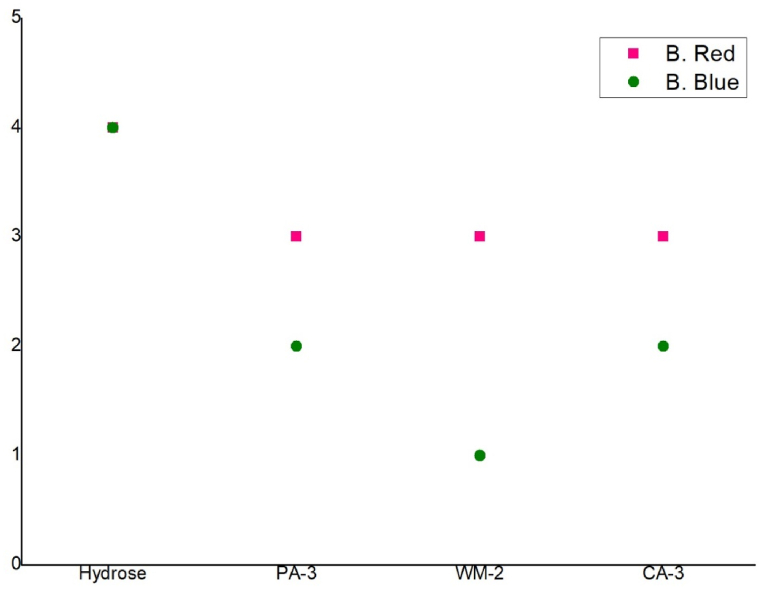
Pilling resistance of treated fabric with different reducing agents.

The pilling properties of the dyed fabrics were a lacking for natural reducing agents compared to hydrose. For both dyes, Fig. 7, hydrose showed slight pilling (4) where all samples for B red dyes showed moderate pilling (3) and for B. Blue, pineapple and carambola showed severe pilling (2). The lowest was for watermelon which was very severe pilling (1). This is a drawback of natural reducing agent which could be reduced by farther treatment.

### 3.8 Ecological performance measurement

Fig. 6 shows the ecological advantages of the natural reducing agents. The highest BOD in Fig. 6a was for hydrose 260 and 255 ppm treated effluent and the lowest was for carambola in B red 200 ppm and watermelon for B blue193 ppm.

The COD values for hydrose in Fig. 6b were 415 ppm and 400 ppm respectively. Whereas pineapple for B red (395 ppm) and carambola for B blue (392 ppm) showed the highest values. Though these values are not as much as hydrose. Among all the natural reducing agents, watermelon showed the lowest COD values which are 375 ppm and 360 ppm respectively for B red and B blue.

In Fig. 6c, the TDS values for hydrose were 2020 and 2040 ppm respectively for both dyes. Where the lowest values from natural reducing agents were 1760 and 1750 ppm for watermelon in both cases. Thought the highest values for reducing agents were for pineapple and carambola as 2nd highest which are not as much as hydrose.

The DO values, Fig. 6d, also showed the opposite trends which means the lowest values for hydrose (4 and 4.5 ppm respectively). Here higher the DO means better the effluent for living bodies. So the pineapple effluent showed the best DO among all samples.

The values of pH of hydrose treated effluents were 12.35 and 12.36 respectively which are very high (Fig. 6e). Although a higher level of basic pH should be maintained during the vatting process with reducing agents the aim of this study was to conduct eco-friendly dyeing. The natural reducing agents, here, showed comparatively little lower values of pH. Which were near 12.25.

All the ecological performance parameters indicate the application of natural reducing agents as a potential eco-friendly process for vat dyeing.

**Fig. 8:**
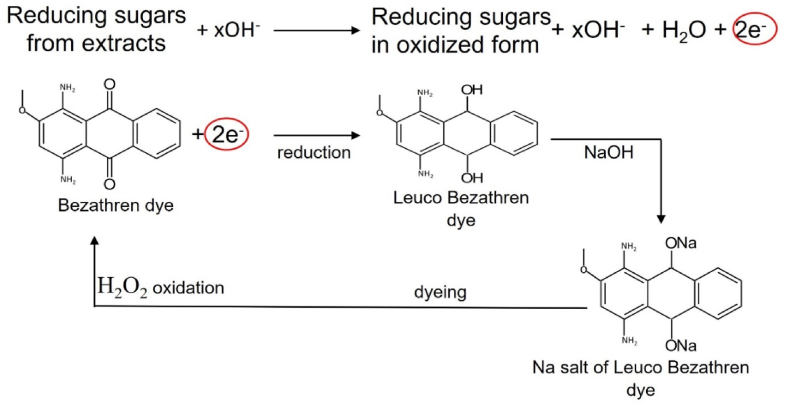
Mechanism of vat dyeing with natural reducing agents.

In Fig. 8, The basic oxidation mechanism for Bezathren dyes is the monosaccharides from the extract which give up electron to reduce the dyes into water soluble form. The water-soluble form then reacts with fabric to form color and finally oxidation is done to make the dye water insoluble again.

## 4 Conclusion

The three natural reducing agents were successfully used in vat dyeing of cotton fabric which showed comparable reducing potentials to hydrose. The FTIR spectra proved the facts also. The dyed fabric showed a good range of colorimetric properties (Lab and Lch), color strength(K/S) as well as color fastness to wash, perspiration and rubbing where carambola showed the best result. The concentration of reducing agents showed best results in an optimum concentration. The bursting strength of dyed fabrics have significantly increased after dyeing where decreased in case of hydrose. Moreover, the ecological performances (BOD, COD, TDS, DO and pH) also showed a better result for natural reducing agents compared to hydrose. However, colorfastness to light and pilling resistance were a little drawback which need more work. All the findings of the study broadens a path for eco-friendly, sustainable, nontoxic and biodegradable alternative dyeing of cotton fabric with natural reducing agents extracted from Pineapple barks, Water melon and Carambola.

## Fund

None.

## Author contribution statement

Md. Hossain Shahid Shrwardi: conceived and designed the experiments; performed the experiments;

Shohag Chandra Das: analyzed and interpreted the data; contributed reagents, materials, analysis tools or data; wrote the paper.

Md. Abdullah Al Mamun: Analyzed and interpreted the data.

## Data availability statement

Data included in article/supplementary material/referenced in article.

## Declaration of competing interest

The authors declare that they have no known competing financial interests or personal relationships that could have appeared to influence the work reported in this paper.

## References

[1] Adeel S., Saeed M., Abdullah A., Rehman F., Salman M., Kamran M., Zuber M., Iqbal M. (2018). Microwave assisted modulation of vat dyeing of cellulosic fiber: improvement in color characteristics. J. Nat. Fibers.

[2] Fox M.R. (1949). The relationship between the chemical constitution of vat dyes and their dyeing and fastness behaviour. J. Soc. Dye. Colour..

[3] Benkhaya Said, rabet Souad M’, Ahmed El Harfi (2020). A review on classifications, recent synthesis and applications of textile dyes. Inorg. Chem. Commun..

[4] Baumgarte U. (1987). Developments in vat dyes and in their application 1974–1986. Rev. Prog. Coloration Relat. Top..

[5] Sutlović Ana, Martinia Ira Glogar, Čorak Ivana, Tarbuk Anita (2021). Trichromatic vat dyeing of cationized cotton. Materials.

[6] Rai Smriti, Saremi Raha, Sharma Suraj, Minko Sergiy (2021). Environment-friendly nanocellulose-indigo dyeing of textiles. Green Chem..

[7] Saikhao Laksanawadee, Setthayanond Jantip, Karpkird Thitinun, Suwanruji Potjanart (2017). Comparison of sodium dithionite and glucose as a reducing agent for natural indigo dyeing on cotton fabrics. MATEC Web of Conferences.

[8] Adane Teshale, Tiruneh Adugna Amare, Alemayehu Esayas (2021). Textile industry effluent treatment techniques. J. Chem..

[9] Teli M.D., Paul Roshan, Landage Sachin M., Arnab Aich (2001). Ecofriendly processing of sulphur and vat dyes - an overview. Indian J. Fiber Textil Res..

[10] Meksi Nizar, Ben Ticha Manel, Kechida Moez, Mhenni Mohamed Farouk (2012). Using of ecofriendly α-hydroxycarbonyls as reducing agents to replace sodium dithionite in indigo dyeing processes. J. Clean. Prod..

[11] Hihara Toshio, Okada Yasuyo, Morita Zenzo (2002). Photo-oxidation and -reduction of vat dyes on water-swollen cellulose and their lightfastness on dry cellulose. Dyes Pigments.

[12] Ibidapo T. Adesanya (1992). Application of redox potentials in the selection of reducing agents for vat dyes. Chem. Eng. J..

[13] Motaghi Zahra (2012). The comparison between a natural reducing agent and sodium dithionite in vat, indigo and sulphur dyeing on cotton fabric. Adv. Mater. Res..

[14] Omender Kr, Chakraborty J.N. (2020). Eco-friendly vat dyeing of cotton using alkaline iron (ii) salt as reducing agent. Tekstilec.

[15] Saikhao Laksanawadee, Setthayanond Jantip, Karpkird Thitinun, Bechtold Thomas, Suwanruji Potjanart (2018). Green reducing agents for indigo dyeing on cotton fabrics. J. Clean. Prod..

[16] Shin Younsook, Choi Min, Dong Il Yoo (2013). Utilization of fruit by-products for organic reducing agent in indigo dyeing. Fibers Polym..

[17] Hossain Md Delwar, Rahman Khan Md Mashiur, Uddin Md Zulhash (2017). Fastness properties and color analysis of natural indigo dye and compatibility study of different natural reducing agents. J. Polym. Environ..

[18] Santhi P., Jeyakodi Moses J. (2008). A study of vat dyeing on cotton fabric assisted by zinc as reducing agent. Orient. J. Chem..

[19] Santhi P., Jeyakodi Moses J. (2010). Study on different reducing agents for effective vat dyeing on cotton fabric. Indian J. Fiber Textil Res..

[20] Moses J Jeyakodi (2023).

[21] Baird Rodger B., Eaton Andrew D., Rice Eugene W. (2017).

[22] Wang Yu-wen, Yi Qing-zhu, Ding Yi, Ji Feng, Wang Ni (2020). Study on the factors influencing the dyeing performance of cotton fabric with vat dyes based on principal component analysis. J. Text. Inst..

[23] Sela Salma Katun, Nayab-Ul-Hossain A.K.M., Islam Rakib Md Shafikul, Khalid Hasan Niloy Md (2020). Improving the functionality of raw cotton: simultaneous strength increases and additional multi-functional properties. Heliyon.

